# Nanoimprint Replication of Biomimetic, Multilevel Undercut Nanostructures

**DOI:** 10.3390/nano11041051

**Published:** 2021-04-20

**Authors:** Michael Muehlberger, Stephan Ruttloff, Dieter Nees, Amiya Moharana, Maria R. Belegratis, Philipp Taus, Sonja Kopp, Heinz D. Wanzenboeck, Adrian Prinz, Daniel Fechtig

**Affiliations:** 1 Profactor GmbH, Im Stadtgut D1, 4407 Steyr, Austria; Amiya.Moharana@joanneum.at (A.M.); Sonja.Kopp@profactor.at (S.K.); Daniel.Fechtig@profactor.at (D.F.); 2 JOANNEUM RESEARCH Forschungsgesellschaft mbH, 8160 Weiz, Austria; Stephan.Ruttloff@joanneum.at (S.R.); Dieter.Nees@joanneum.at (D.N.); Maria.Belegratis@joanneum.at (M.R.B.); 3 Institute for Solid State Electronics, TU Wien, 1040 Vienna, Austria; Philipp.Taus@tuwien.ac.at (P.T.); Heinz.Wanzenboeck@tuwien.ac.at (H.D.W.); 4 Stratec Consumables GmbH, 5081 Anif, Austria; A.Prinz@stratec.com

**Keywords:** nanoimprint lithography, UV-NIL, biomimetics, morpho butterfly

## Abstract

The nanoimprint replication of biomimetic nanostructures can be interesting for a wide range of applications. We demonstrate the process chain for Morpho-blue-inspired nanostructures, which are especially challenging for the nanoimprint process, since they consist of multilayer undercut structures, which typically cannot be replicated using nanoimprint lithography. To achieve this, we used a specially made, proprietary imprint material to firstly allow successful stamp fabrication from an undercut master structure, and secondly to enable UV-based nanoimprinting using the same material. Nanoimprinting was performed on polymer substrates with stamps on polymer backplanes to be compatible with roller-based imprinting processes. We started with single layer undercut structures to finally show that it is possible to successfully replicate a multilayer undercut stamp from a multilayer undercut master and use this stamp to obtain multilayer undercut nanoimprinted samples.

## 1. Introduction

In the course of evolution, nature has developed a broad range of nanostructured surfaces with many different purposes: to optimize friction behavior (e.g., [1,2,3]) or the behavior of liquids (e.g., [4,5]), to achieve antimicrobial (e.g., [6,7]) or antireflective effects (e.g., [8,9,10]) and to achieve decorative effects (e.g., [11,12,13,14]), just to name a few. Many of these nanostructures are also of high interest for technical applications and have been investigated accordingly (e.g., [1,15,16,17]). 

Nanoimprinting [18,19,20] is a replication technology, capable of replicating nanoscale (e.g., [21,22]) as well as microscale features [23] on large areas [24,25,26]. 

Due to the fact that nanoimprinting can—in a single process step—replicate complex multilevel structures [1,24,27,28], it is ideally suited for the replication of such biomimetic or bio-inspired nanostructures. A big challenge for nanoimprinting, however, is the replication of undercut features, since it is usually not possible to remove the stamp from such features, especially if they are of nanoscale. It has, however, been shown in the literature that this can be achieved for larger features in the several µm range [29,30].

The nanostructures we are interested in are responsible for the metallic blue color of the Morpho butterfly. These structures have attracted considerable interest and their functioning has been researched in detail [31,32]. Based on the understanding of the optical properties of these nanostructures, it has been possible to mimic the optical effect using nanostructured surfaces and multilayer stacks of deposited materials [31]. We wanted to investigate if it might be possible to avoid such complicated and expensive partly vacuum-based processes and replace them with a single nanoimprint process. The aim of the work presented in this paper was, however, not to replicate the full optical effect, but to demonstrate the basic possibility. The mastering process would have been significantly more complicated if we would have included the necessary randomness to its full extent as it would have been necessary to replicate the full optical effect. The capabilities of the nanoimprint process can, however, also be shown with a reduced master design.

## 2. Materials and Methods 

### 2.1. Master Design and Fabrication

**Figure 1:**
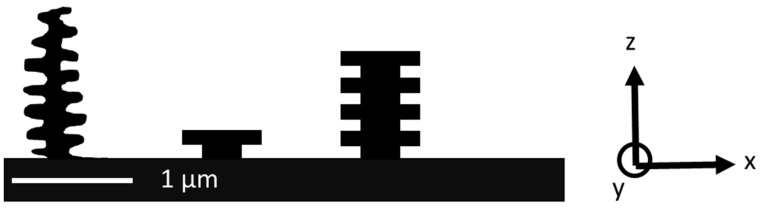
Left: sketch of the cross-section of the nanostructures responsible for the metallic-blue color of the Morpho butterfly. The sketch was created after images found, e.g., in [12,33]. Center and right: model structures used in this work. We started with a single layer “T-structure” and continued to work with the more complex “tree-structure”.

Figure 1 schematically shows cross-sections of the structures that we dealt with in this work. The very left structure represents the structure as it is found in nature and is drawn after SEM images as they are found in the literature (e.g., [12,33]). For our purpose, we repeated the individual structures periodically along the x-direction. The structures extend along the y direction, as can also be seen later in Figure 6 (left image).

As far as we understand from the literature [34], to obtain the full optical effect of the Morpho butterfly in our model structures, several aspects are missing, most notably randomness in the lateral placement of the individual “trees” as well as randomness in the height of the “trees”. Furthermore, the branches of the trees are not positioned at the same height on the left and on the right side of the trunk in the real Morpho butterfly structure. Taking these facts into account for our master design would have significantly increased the challenges in master fabrication. At the same time, the nanoimprint challenge would have been basically the same. Therefore, since the goal was to investigate if nanoimprinting can be used to replicate such a structure, we focused on the model structures as sketched in Figure 1 in the center and on the right and did not include the randomness. 

Details on the masters and the mastering process are described in [35]; here, only the basic facts should be mentioned. All masters were fabricated on silicon wafers, first using a multilayer deposition followed by lithography and anisotropic etching to etch through the multilayer stack. Additionally, one of the layer materials was selectively etched to create the undercut. The layer heights of the multilayer stack were 85 nm and 50 nm. The lateral width of the single T-structure was around 400 nm after processing, while tree structures with different lateral dimensions were fabricated. Undercuts in the range of 100 nm were typically achieved. 

### 2.2. Nanoimprint Material

The imprint resin primarily used in this work is an in house development of JOANNEUM RESEARCH Forschungsgesellschaft mbH. In the development process, 13 different acrylate resins have been formulated and tested, with the main focus on low viscosity, high elasticity and low surface energy in order to enable the fast and complete filling of the undercut features and easy demolding of the cured structures. Some of these resins had problems during demolding and stuck to the stamp, e.g., leaving residual material in the undercut areas. Others demolded easily but did not replicate the undercut features. Typical defects were collapsed T-structures. Out of the tested ones, the resin named NILcure^®^ JR5 (JOANNEUM RESEARCH, Weiz, Austria) proved to be optimal for high fidelity replication of undercut structures. 

This elastic UV curable acrylate resin can be efficiently cured within seconds by UV-A light between 350 nm and 400 nm at a moderate irradiance of 50–100 mW/cm^2^. Additional properties facilitating the UV imprinting of undercut features are low viscosity (<20 m Pas), low glass transition temperature (below room temperature), high crosslinking and low surface energy after curing down to 20 mN/m—depending on the surface energy of the stamp [25]. Furthermore, this imprint material shows high scratch and abrasion resistance as well as chemical and weathering stability. 

A key feature of NILcure^®^ JR5 that was used in the course of this project is also that it can be used as a stamp material for NILcure^®^ JR5 itself, meaning that it is self-replicable. The nanoimprint process using this material is described in the next section.

The challenge was to find the right balance between surface energy and mechanical properties, i.e., Young’s modulus, tensile strength and elongation at break of the cured imprint resin. The surface energy of the resins was tuned by doping the resins with highly surface active anti-adhesive additives—such as 1H,1H,2H,2H-perfluorooctyl-acrylate—as well as a JR-proprietary related molecule. 

For tuning the viscosity of the uncured and the mechanical properties of the cured imprint resins, various formulations containing multifunctional acrylate oligomers with aliphatic poly-urethane and poly-siloxane backbones as well as mono- and bifunctional acrylate monomers were investigated. The acrylate oligomers were chosen according to their mechanical properties, ranging from Young’s Modulus = 20 MPa–100 MPa, tensile strength = 5–25 MPa and elongation at break = 10–100%. For formulating the UV-imprint resins, reactive diluents had to be added to the highly viscos acrylate-functionalized oligomers. Mono- as well as bi-functional acrylate monomers were chosen. Whilst the addition of mono-functional acrylate monomers largely preserves low Young’s modulus and high elongation at break, the tensile strength is generally lowered. The addition of bifunctional acrylate monomers, on the other hand, decreases the elongation at break and increases the Young’s modulus and tensile strength of the cured resins [36,37].

NILcure^®^ JR5 featuring optimal mechanical properties and the JR proprietary fluorinated anti-adhesive additive turned out to enable the highest imprint fidelity for the undercut structures of all investigated resin formulations.

**Figure 2:**
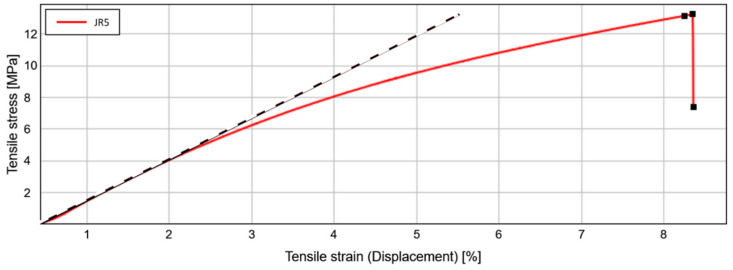
Tensile stress plotted versus tensile strain for the NILcure^®^ JR5 material.

A universal testing machine (Instron 3342 [38]) was used to determine the mechanical parameters of the most successful material NILcure^®^ JR5 (see Figure 2). This machine can be used to perform classical tensile tests (Young’s modulus, tensile strength, elongation at break) as well as other important measurements such as friction and adhesion measurements (peel test). For the determination of the Young’s modulus and the elongation at break (e.g., [39]), a classical tensile test was performed. The rectangular tensile specimens had dimensions of 40 mm × 10 mm × 150 μm (clamping length, width, thickness). The test speed was 5 mm/min. The following figure shows an exemplary measurement of JR5. The following parameters were obtained: Young’s modulus: 246 MPa, elongation at break: 8%. The surface energy of the liquid material is 25.5 mN/m as determined by the pendant drop method [40,41], using a DSA100 from Krüss [42].

Initially, materials such as h-PDMS (hard Polydimethylsiloxane) [43] and MD700 [44] as stamp materials and OrmoComp [45] as an imprint material were used. However, the results were not encouraging in terms of defectivity (see later in Figure 4), which is why our own materials were formulated and tested. The results are discussed below in the results section.

### 2.3. Nanoimprint Process

**Figure 3:**
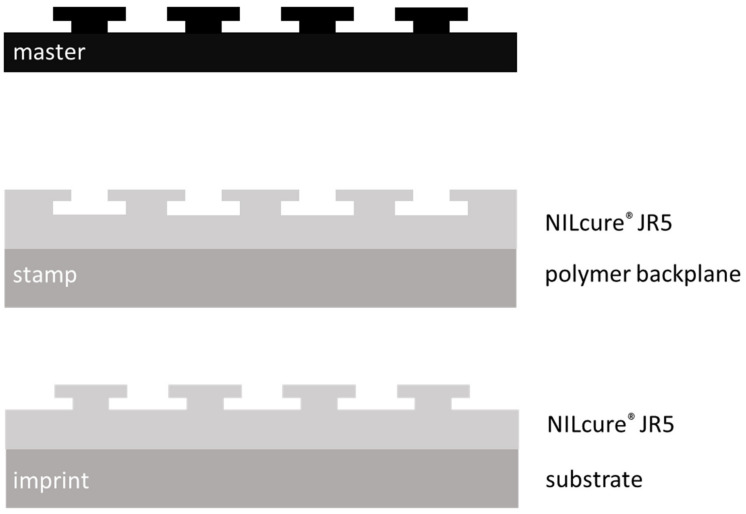
Sample sequence and nomenclature for this work: From a master, a nanoimprint stamp was fabricated (stamp material on backplane), which was then used to create the imprint (imprint material on substrate).

The goal of our nanoimprint process was to replicate a complex undercut nanostructure in a single imprinting step. To do so, we fabricated a stamp from a nanoimprint master and used this stamp in a nanoimprint process. For all process steps, we used UV-based nanoimprinting, i.e., UV-curable materials. The sample fabrication sequence is sketched in Figure 3.

All masters were treated with an anti-sticking layer (BGL-GZ-83 from PROFACTOR GmbH [46,47]) to allow for easy removal of the stamp from the master structure. To fabricate the stamp, NILcure^®^ JR5 resin was manually deposited on the master. A UV-transparent flexible backplane was used and placed gently onto the droplet in a rolling motion alongside the direction of the T-structures (y-direction in Figure 1). The whole stack was UV-exposed with a wavelength of 365 nm and an intensity of about 100 mW/cm^2^ for a minimum of 30 s. Both UV-LED (light emitting diode) light sources (self-made at PROFACTOR, 365 nm) and UV fluorescent tubes (supplier: biostep GmbH [48]) can be used. After curing, the stamp was very gently removed from the master, again in the y-direction, to ensure easier demolding and also to prevent the tree branches from being destroyed.

As backplanes, different polymer films have been used. Two hundred micrometer-thick commercial PVC (polyvinyl chloride) films worked well, as well as PET (polyethylene terephthalate) films with a thickness of 125 µm. The PVC was treated with an oxygen plasma before applying an adhesion promoter (HMNP12 from PROFACTOR GmbH [49]). The PET films were already supplied with an adhesion promoter, such as Kemafoil HSPL80 from Coveme [50,51] and Melinex ST506 from DuPont [52]. No backplane was used for the imprints using h-PDMS stamps.

After having obtained the stamps in the way described above, the imprints were made using those stamps in the same way. The imprint material was manually placed on the substrate, the stamp was carefully brought into contact with the coated substrate and, after curing, the stamp was separated from the finished imprint. The substrates that were used were the same polymer foils as for the backplanes for the stamps, but also glass substrates were used (e.g., Superfrost Ultra Plus [53]). 

For both processes—the stamp fabrication as well as the actual imprinting—no additional pressure was applied. The imprinting was performed manually and was left to the capillary forces to obtain good contact between the stamp and substrate. The imprinting area was always several mm^2^, typically 5 × 5mm^2^.

There are two reasons for using polymer foils in this process chain. Firstly, it was necessary for sample characterization for both stamps and imprints, as described below. Secondly, and this is only valid for the stamp, the flexibility of the stamp allowed for easy separation of the stamp from the imprint in a peeling-like motion. The flexibility and UV-transparency will also allow the use of such stamps as imprinting plates in roller-based nanoimprint processes [25,26], which would pave the way for industrial implementation.

### 2.4. Sample Characterisation

Sample characterization was performed using atomic force microscopy (AFM) (Bruker Dimension Edge, Bruker Corporation, Billerica, MA, USA) (PROFACTOR) and scanning electron microscopy (TU Wien (Zeiss Neon 40 EsB (Zeiss Microscopy, Jena, Germany) and JOANNEUM RESEARCH (SM-IT 100 (JEOL, Tokyo, Japan)). For the characterization of the master, focused ion beam (FIB) cutting (TU Wien using a Zeiss Neon 40 (Zeiss Microscopy, Jena, Germany) was also used. It proved to be challenging to obtain reliable information on the undercut on the polymer samples from these methods. AFM imaging naturally cannot provide direct evidence of any undercut features, but also indirect evidence gathered by comparing the lateral dimensions of master, stamp and imprint proved to be not conclusive. One reason for this is tip convolution effects. Additionally, standard SEM imaging caused problems since the polymeric nanostructures tend to deform under electron beam bombardment or during FIB cutting. 

High-quality cross sections of the nanostructures (in x-direction) on polymer substrates were made by means of cryo-ultramicrotomy (PowerTome XL RMC Products, Boeckeler^®^, Tucson, AZ, USA [54]). The samples were physically hardened by cooling with liquid nitrogen below their glass transition temperature at −60 °C. Nanomaterial imaging and analysis were performed by utilizing the scanning electron microscope RAITH e-line at 3 kV. Cryo-ultramicrotomy at JOANNEUM RESEARCH for sample preparation proved to be the only variant that led to reliable results.

## 3. Results

### 3.1. Single Layer Undercut Structures

Using the imprinting and characterization procedures described above, we were able to show the full nanoimprint chain from master to final imprint, for both the single “Ts” as well as the “trees”.

**Figure 4:**
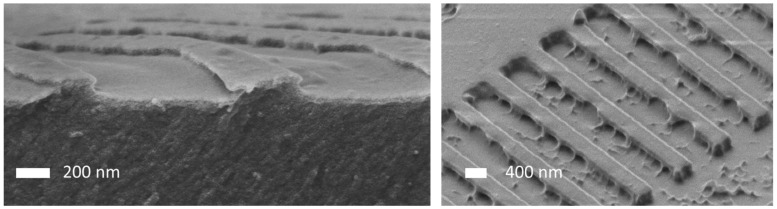
Left: cross-sectional SEM image of an imprint using an h-PDMS stamp and OrmoComp as imprinting material. Right: SEM image imprint using MD700 as stamp material and OrmoComp as imprint material.

**Figure 5:**
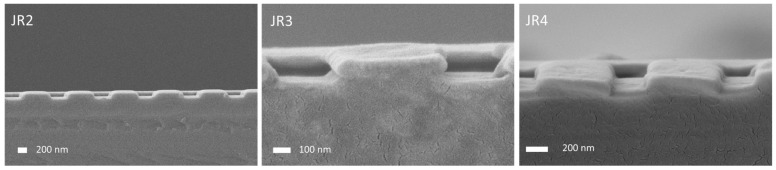
Cross-sectional SEM images of stamps made from different materials. For NILcure^®^ JR2 and JR4 it can be seen that the undercut is not replicated at all, while for JR3 the undercut is only partly replicated.

In the course of the project, and before the NILcure^®^ JR5 material was used, several other material combinations were tested. Results are shown in Figure 4 and Figure 5. Initially, our approach was to use soft stamps and more rigid imprint materials, such as h-PDMS or MD700 as soft stamp materials and OrmoComp as an imprint material. However, due to the limited tuning possibilities with these commercial materials and the high number of defects (see Figure 4) observed already in the stamps for our special application, we started to test our own materials. In Figure 4, two different material combinations are shown. On the left, the result of an imprint using an h-PDMS stamp and OrmoComp as the imprinting material is shown. As can be seen, the T-shapes are not well defined and the edges of the Ts are rough and irregular, indicating problems during the imprinting step—either non-filling of the undercut or mechanical tear-off of the overhanging part of the nanostructures, or possibly both. A lot of defects can also be seen in the right image of Figure 4, which shows an SEM image of an OrmoComp imprint using an MD700 stamp. Additionally, here the overhanging structures are not well replicated. Here, defects on the bottom can also be observed, which are, however, related to the defective master which was used for these experiments and are not related to the undercut structures. 

Switching to the self-made materials described above initially resulted in stamps which did not replicate the undercut, either completely or only partly, as shown in Figure 5, where cross-sectional SEM images of three stamps made from three different experimental materials are shown. It can be seen that the undercut is not replicated (left image and right image) at all, or only partly (center image).

The images in Figure 4 and Figure 5 give a representative overview of the typical defects we observed: either non-replication of the undercut or only partial replication. We attribute the non-replication of the undercut either to non-filling of the undercut part of the structures, which is related to the wetting properties of the material and its viscosity, or to a material which was too soft, so that the nanostructures collapsed. The second type of defect we observe is when the undercut was only partly replicated. This we attribute to an imprint material that was mechanically too brittle to allow for a defect-free separation. The defects described above appear on the whole area with the same density, meaning that the full imprint area cannot be used. This is in contrast to other typical nanoimprint defects induced by dust particles or air bubbles, which are very local. In summary, the mechanical stability of the stamp and the imprint material have to be right as well as the surface energy and wetting properties.

**Figure 6:**
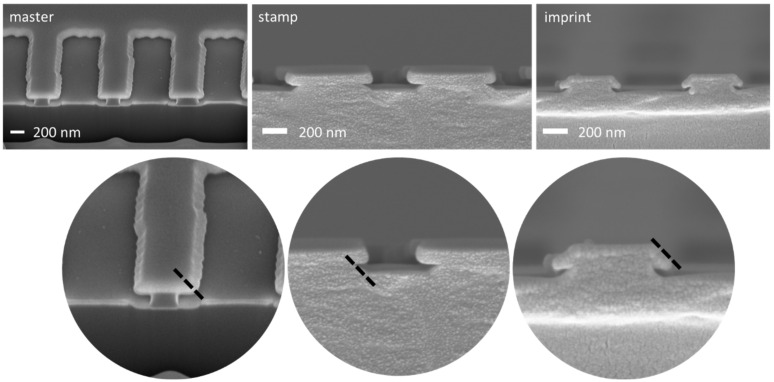
SEM cross-sectional images of the sample sequence from the master structure [35] (left) over the stamp in NILCure^®^ JR5 (center) and the imprinted structures in NILcure^®^ JR5 (right). The second row shows close-ups from the images in the upper row.

The material that fulfilled all requirements was the NILcure^®^ JR5 material decribed above. Figure 6 shows SEM images of the full sequence from master over stamp to imprint using the NILcure^®^ JR5 nanoimprint material. The left images show the master structure, the center images the stamp, which was replicated from the master, and to the very right the imprint is shown, which was made using the stamp. It can be seen that the undercut features are nicely replicated. Looking at the master structure in more detail, a slight tapering of the T crossbar can be noticed, which is indicated by the dashed line in the second row of images in Figure 6. The same tapering can be observed in the SEM cross-section of the stamp (top-down) and the imprint as well. This shows that in the whole process chain the replication of the undercut structures works very well.

**Figure 7:**
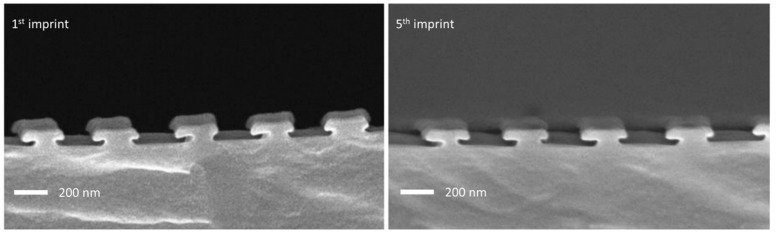
SEM cross-sectional images of the first imprint from (left) the master structure and the fifth imprint (right) using the same stamp.

We successfully used the same stamp for several imprints. Figure 7 shows SEM cross-sectional images of the first imprint (left) and of the fifth imprint (right), all made with the same stamp. This shows that the stamp remains intact during the imprinting process, which is of course a necessary prerequisite for a nanoimprint process. The difference in the shape of the edges of the T-structures compared to the ones shown in Figure 6 results from the fact that a different master was used, which exhibited a slightly different geometry.

### 3.2. Mulitlayer Undercut Structues

**Figure 8:**
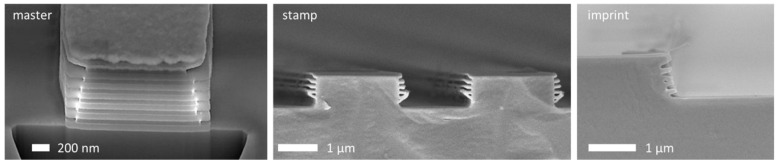
SEM cross-sectional images of the multilayer structure sequence from the master structure in Si (left) over the stamp in NILCure^®^ JR5 (center) and the imprinted structures in NILcure^®^ JR5 (right).

In addition, the multilayer structures could also be replicated very well with the described procedure. Figure 8 shows the sequence from the master over stamp to the imprint for the multilayer structures. Especially in the images of the stamp and the imprint, it can be seen that the tree branches partially stick together, and this can be attributed to the flexibility of the imprint in connection with the small size of the structures. It cannot be excluded that this is also an artifact of SEM imaging or sample preparation. Nevertheless, it can be seen that the nanoimprint replication of such complex features is possible.

## 4. Discussion

Taking the Morpho butterfly structure as an example, we could show that nanoimprinting is capable of replicating complex undercut nanostructures. For such a process to work, it is crucial to find a material which exhibits the right wetting properties as well as suitable mechanical properties. This broadens the potential of nanoimprinting significantly and opens up the possibility for a cost effective single-step replication of, e.g., bioinspired Morpho-blue nanostructures. We tested several material combinations to arrive at a process–material combination that reliably allowed for the replication of such structures. Although all the results shown in this paper were obtained using manual nanoimprinting, we designed our processes in such a way that the transfer to roller-based processes [25,26] is possible. The roller-based nanoimprint processes will also mimic the peeling-like separation of the stamp from the imprint, which was used successfully in our experiments.

Further research will be necessary to see how the material formulation and material properties and the dimensions of the undercut nanostructures are related, i.e., if the same material can be used for significantly larger or smaller structures. The research presented here is, in our opinion, a first important step since it shows for the first time that the replication of undercut nanostructures is feasible using UV-based nanoimprint lithography.

Regarding the Morpho butterfly structure, it will be interesting to introduce the necessary randomness in the master design to faithfully reproduce the optical effects. Using nanoimprinting, it then should be possible to replicate these structures in a single imprinting step in a cost efficient way. This will be facilitated in more automated processes which will also reduce defectivity. 

Depending on the application, it might be interesting or necessary to protect the imprinted nanostructures, which would require a material with a proper refractive index contrast to the imprinting resin—a topic which we did not address so far. 

Furthermore, we hope to come across many more interesting nanostructures, for all types of applications, which could be enabled using this or similar processes. 

## Data Availability

The data presented in this study are available on request from the corresponding author.
